# Exploring Hajj pilgrim satisfaction with hospitality services through expectation-confirmation theory and deep learning

**DOI:** 10.1016/j.heliyon.2023.e22192

**Published:** 2023-11-10

**Authors:** Marwan Albahar, Foziah Gazzawe, Mohammed Thanoon, Abdulaziz Albahr

**Affiliations:** a Department of Computer Science, Umm Al Qura University, P.O. Box 715, Mecca, Saudi Arabia; b College of Applied Medical Sciences, King Saud bin Abdulaziz University for Health Sciences, Alahsa, Saudi Arabia; c King Abdullah International Medical Research Center, Alahsa, Saudi Arabia

**Keywords:** Artificial neural networks, Hajj, Satisfaction, Hospitality services, Deep learning model

## Abstract

The Hajj is a religious event that attracts a significant number of Muslims from various countries who perform rituals in Mecca, Saudi Arabia. Despite the high volume of pilgrims that typically participate in the event, the number has been reduced in recent years due to the COVID-19 pandemic. The satisfaction of Hajj pilgrims with the quality of hospitality services provided during the event is a crucial factor that must be studied and understood. To achieve this goal, various psychological theories have been employed to explain the phenomenon. The advancement of big data and artificial intelligence has enabled the development of new analytical methodologies for evaluating psychological theories in the hospitality industry. In this study, we present a novel deep learning model that leverages the expectation-confirmation theory to examine the satisfaction of Hajj pilgrims with hospitality services. The model was trained and tested on data obtained from hotel review posts related to the Hajj. Based on our results, the proposed model achieved a high accuracy of 97 % in predicting the satisfaction of Hajj pilgrims. In addition, the results can be used to improve the quality of services provided to pilgrims and enhance their overall experience during the Hajj.

## 1 Introduction

Customer satisfaction is an essential metric that measures how well a product or service meets or exceeds customer expectations. It is a complex process that considers factors such as quality, price, and service. Satisfied customers are more likely to become loyal and repeat customers, which can lead to reduced price sensitivity and increased protection against competitors. Additionally, customer satisfaction can help reduce future transaction costs, increase reputation, and attract new customers [1].

Scholars worldwide are increasingly interested in studying the intersection of tourism and religion. While earlier research primarily focused on anthropological and sociological aspects [2,3], more recent studies have also examined geological and historical factors [4,5]. Religion can be a significant motivator for travel and is responsible for many captivating tourist destinations [6]. Thanks to advances in communication and transportation, travel to religious sites has become more accessible. Pilgrimage to various locations provides significant economic, social, and political benefits and has evolved to be more than just a religious journey; it can be a transformative, secular, or non-religious experience too [7]. Christianity, Judaism, Hinduism, and Islam all have pilgrimage traditions, with the Hajj to Mecca being one of the most well-known examples. During the Hajj, muslim pilgrims embark on a journey to four significant destinations, namely Mecca, Arafat, Muzdalifa, and Mina. The Ministry of Pilgrimage organizes the Hajj's journey, with oversight from the Supreme Hajj Committee [8]. The annual Hajj pilgrimage, which is a gathering of Muslims, is effectively managed by collaborating among numerous officials and ministries, including those in information and culture, health, telecommunications, and information technology, in addition to the Saudi Red Crescent and the National Guard. Furthermore, the participation of private and non-tourism industries can play an essential role in increasing the capacity of transportation, accommodation, and other amenities to pilgrims. However, the management of a vast number of pilgrims presents a unique logistical challenge, necessitating the use of advanced technologies in traditional practices that have been in place for years [9]. To address these challenges, a tremendous number of buses are utilized on the superhighway connecting Jeddah and Mecca to transport pilgrims to dedicated, air-conditioned tents in Mina. The pilgrimage sites at this location are well-equipped with an enormous number of drinking fountains, medical facilities, and telephone banks. Nevertheless, in spite of these arrangements, pilgrims might face potential hazards and risks. The main aims of the Pilgrim Experience Program are to make it easier for pilgrims to access sacred places, provide superior services, and enrich their cultural and spiritual experiences [10]. To achieve these goals, research and studies should be conducted to investigate current issues, improve positive aspects of service quality, and take the spirituality of pilgrims into account. Factors that may impact pilgrims' spirituality, such as their level of satisfaction with the quality of services provided during Hajj, should be considered. Pilgrims' perceptions of their overall experience of the services provided by Hajj packages may influence their spiritual experience. Therefore, Saudi Arabia's national authorities should consistently oversee the operational procedures for religious tourists and the caliber of services rendered to enhance their satisfaction and overall experience. Previous studies have shown that Hajj organizers worldwide, including those in Saudi Arabia, strive to enhance service quality to ensure pilgrims satisfaction [6].

Nonetheless, most studies on Hajj events provide only limited analysis and interpretation of pilgrims' data. Moreover, these studies lack a distinct framework for examining the factors contributing to pilgrim satisfaction and connecting the outcomes with different events during the Hajj. As a result, the obtained outcomes are restricted to a linear correlation, rendering it challenging to visually determine the extent of diverse factors that impact one another in the pilgrims' satisfaction data.

To address such problem, this study proposes a deep learning technique that is based on new regularization to unveil hidden correlations and significant insights from intricate multidimensional data. The theory of expectation-confirmation is used to explain customer satisfaction within the hospitality industry. The goal of this study is to establish a mechanism for analyzing and interpreting pilgrims' satisfaction. Comparing to previous works, our work not only detects various associations among factors impacting pilgrims' satisfaction but also evaluate the performance against other machine learning and deep learning methods. Moreover, to the best of our knowledge, this is one of the limited attempts to apply deep learning techniques for assessing pilgrims' satisfaction.

This paper is organized as follows. Section 2 discusses the related works. The proposed approach and dataset description are presented in Section 3. The detailed results are presented in Section 4. Discussion and limitations are presented in Sections 5, 6, respectively. Finally, conclusions are drawn, and future directions are discussed in Section 7.

## 2 Related works

The hospitality and tourism industries are utilizing advanced technologies such as robotics and artificial intelligence to enhance customer service and experiences. These technological advancements have led to the emergence of smart tourism, which presents new opportunities for research. The hospitality sector is known as the most competitive edge market globally, also according to previous studies regarding this sector, consumer behavior research has remained a prominent issue in this area. According to Refs. [11,12] various studies have thus been carried out to analyze consumer behavior from all possible aspects for the success of the industry. Customer satisfaction is specifically considered a significant determinant of loyalty and has been considered in the research work of the hospitality industry [13,14]. Additionally, more recent studies investigate customer satisfaction from online reviews through comments to obtain knowledge about customer needs and behavior [15,16] while also confirming and improving their experiences [17,18].

Big data has become a popular tool in modern technology that can significantly enhance the consumer experience and aid customers in making informed decisions about their purchases [19]. The hospitality industry, which is highly competitive, has seen a growing number of hoteliers adopting big data to generate additional value for their customers [20]. By utilizing big data, hotels can create personalized products and services that differentiate them from their rivals [21]. Moreover, big data provides businesses with the ability to explore unforeseen patterns in customer behavior, market trends, and company practices, ultimately leading to a better comprehension of their customers, a crucial element for success in the hospitality sector [22]. Deep learning has recently been applied to various aspects of the hospitality sector, such as customer satisfaction, service quality, and employee performance. For example, in a recent study by Chen et al. [23], a deep learning model was used to predict customer satisfaction with hotel services based on online reviews and ratings. The authors found that the trained model was able to accurately predict customer satisfaction and identify the most important factors that contributed to it. This study demonstrated the potential of deep learning for understanding and improving customer satisfaction in the hospitality industry. Another study by Kim et al. [24] used deep learning to predict the quality of hotel services based on customer feedback. The authors trained a deep neural network on a dataset of customer reviews and ratings and evaluated its performance in predicting service quality. They found that the trained model was able to accurately predict service quality and identify the most important factors that contributed to it. This study showed the potential of deep learning for predicting and improving service quality in the hospitality industry. In addition, a study by Li et al. [25] used deep learning to predict the performance of hotel employees based on their interactions with customers. The authors trained a deep neural network on a dataset of employee-customer interactions and evaluated its performance in predicting employee performance. They found that the trained model was able to accurately predict employee performance and identify the most important factors that contributed to it. This study demonstrated the potential of deep learning for predicting and improving employee performance in the hospitality industry. In addition to the studies mentioned above, there are many other examples of the use of deep learning in the hospitality industry. For example, a study by Ehsan et al. [26] used deep learning to predict customer satisfaction of airline passengers based on their feedback and interactions with the airline. The authors trained a deep neural network on a dataset of customer feedback and evaluated its performance in predicting customer satisfaction. They found that the trained model was able to accurately predict customer satisfaction and identify the most important factors that contributed to it. This study showed the potential of deep learning for predicting and improving customer satisfaction in the airline industry.

Another study by Zhang et al. [27] used deep learning to predict the demand for hotel rooms based on historical data and customer preferences. The authors trained a deep learning model on a dataset of hotel bookings and evaluated its performance in predicting demand. They found that the trained model showed high accuracy in predicting the demand and identifying the most important factors that contributed to it. This showed the potential of leveraging deep learning techniques for predicting and optimizing demand in the hotel industry. Furthermore, a study by Lee et al. [28] used deep learning to improve the personalized recommendation of hotel services to customers. The authors trained a deep learning model on a dataset of customer preferences and interactions with the hotel and evaluated its performance in recommending services. They found that the trained model was able to accurately recommend services and improve customer satisfaction. This study showed the potential of deep learning for personalizing recommendations and improving customer satisfaction in the hotel industry. In Ref. [29], the authors used deep learning to examine pilgrims' and others' impressions of Hajj in 1442 AH. They collected 4300 Hajj-related tweets and YouTube comments and employed CNNs and LSTM deep learning techniques to study people's perceptions. CNN-LSTM outperformed other classification models in F-score and accuracy.

Generally, these recent studies have shown the potential of deep learning for improving various aspects of the hospitality industry, such as customer satisfaction, service quality, and employee performance. By applying deep learning techniques to large datasets of customer feedback and interactions, hospitality companies can gain valuable insights into the factors that drive customer satisfaction and service quality. Overall, these studies demonstrate the wide range of applications of deep learning in the hospitality industry, including customer satisfaction prediction, service quality prediction, employee performance prediction, demand prediction, and personalized recommendation. By leveraging the power of deep learning, hospitality companies can gain valuable insights into the factors that drive customer satisfaction and service quality and use this information to improve their operations and services. However, the stated reviews by customers after using a specific service may not provide a comprehensive representation of the entire experience of the service, that hinders the deep analysis of the overall customer experience of a service. To this end, we propose a deep learning model that integrates a new regularization and deploys expectation-confirmation theory to analyze the perceptions and satisfaction of pilgrims in the hospitality industry.

## 3 Methodology

### 3.1 Dataset collection and pre-processing

In this study, the data was collected from Booking.com of pilgrim reviews during Hajj season. The data collection relied on a Qualitative method because it is fast and highly efficient, and researchers can reach results in an obvious way using unbiased statistics. The sample also is focused, and its purpose is determined from the start of the study design. In addition to generalizing the results, the test sample was carefully selected [30]. The dataset contains 15,859 reviews written in both modern standard Arabic and dialectal Arabic. Each review includes several attributes, such as the name of the hotel, the rating given by the reviewer on a scale of 1–5, the type of user (single, family, couple), number of nights stayed, room type, and the review text itself. Before using this data to build our proposed model, preprocessing procedure was performed on the dataset. This involves removing any hashtags (#), numbers, URLs, mentions (@), non-Arabic words, and other irrelevant content from the collected tweets. However, the Arabic language is complex, and each word can have multiple forms while still conveying the same meaning. This is because of the rich morphology of Arabic. To overcome this challenge, we prepared the text data using specific techniques to reduce noise and normalize the dataset, as outlined in Table 1.

• Removing any irrelevant or duplicate data.
• Removing any special characters or punctuation from the text data.
• Tokenizing the text data, i.e., breaking it down into individual words or phrases.
• Normalizing the text data, i.e., converting it to a standard format such as lowercase.
• Removing stop words, i.e., common words that do not provide any useful information.
• Stemming the text data, i.e., reducing words to their base form.

**Table 1:** Description of statistic of the dataset.

Class	Training	Testing	Validation
Positive	4301	2100	1009
Negative	2459	1820	922
Neutral	1900	749	599

To enhance the accuracy and efficiency of the model, the pre-processing stage is designed to address the complexities of the Arabic language. This includes removing redundant tokens and reducing the vocabulary size to simplify the language. These steps can help the model perform better and yield more accurate results. In addition to the preprocessing procedure, we conducted extensive statistical analysis on the collected dataset to gain deeper insights into the pilgrim reviews during the Hajj season.

**Fig. 1:**
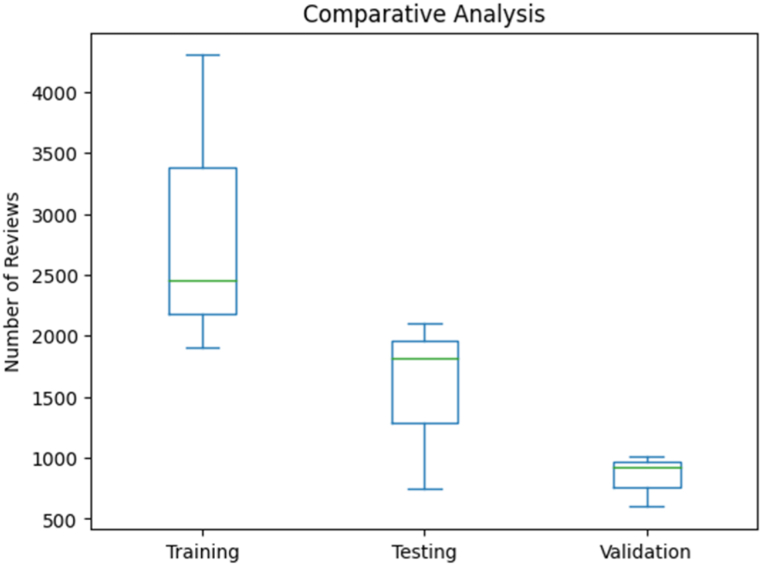
Comparative analysis - distribution of reviews across sentiment classes.

Comparative Analysis: The comparative analysis was conducted to gain insights into the distribution of reviews among different classes (Positive, Negative, Neutral) in the dataset pertaining to pilgrim reviews during the Hajj season (see Fig. 1). Through the utilization of box plots, we visually examined the dispersion of the number of reviews for each class in the training, testing, and validation sets. The results indicated that the Positive class exhibited the highest median number of reviews in both the training and testing sets, suggesting a prevailing tendency of pilgrims to express positive sentiments in their reviews. Conversely, the Negative class displayed the lowest median number of reviews, indicating a relatively lower occurrence of negative sentiments. The Neutral class manifested intermediate median values, suggesting a balanced distribution of neutral sentiments. This comparative analysis provided valuable insights into the sentiment distribution among the classes, guiding potential avenues for further investigation and targeted enhancements in hospitality services to cater to pilgrims' needs effectively.

**Fig. 2:**
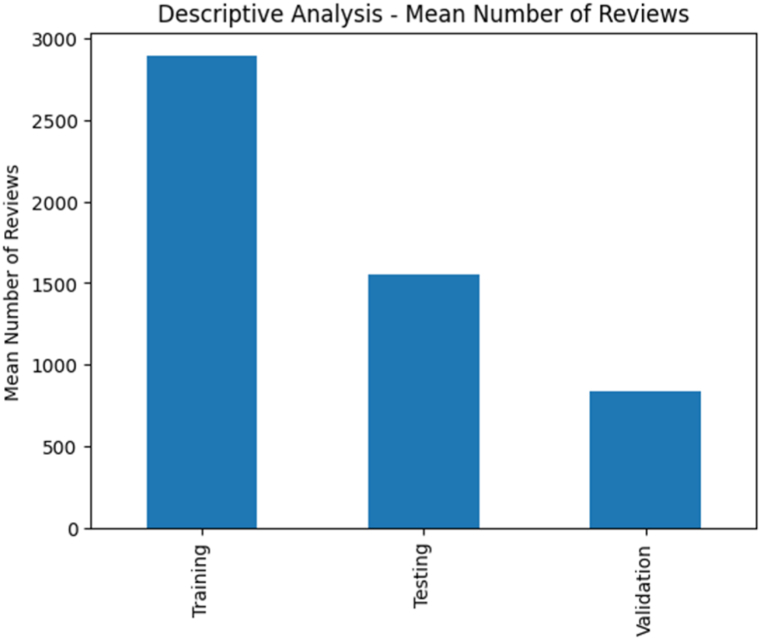
Descriptive analysis - mean number of reviews across sentiment classes.

Descriptive Analysis (Mean Number of Reviews): The descriptive analysis focused on the mean number of reviews for each class (Positive, Negative, Neutral) within the dataset encompassing pilgrim reviews during the Hajj season (see Fig. 2). By computing the mean values, we gained a comprehensive understanding of the average number of reviews submitted by pilgrims, reflecting their sentiments across different classes. The analysis unveiled that the Positive class displayed the highest mean number of reviews in both the training and testing sets, indicating a proclivity among pilgrims to articulate positive sentiments in their reviews. Conversely, the Negative class demonstrated the lowest mean number of reviews, signifying a comparatively lower occurrence of negative sentiments. The Neutral class showcased an intermediate mean, denoting a balanced representation of neutral sentiments. The descriptive analysis rendered invaluable insights into the prevalence of sentiments within pilgrim reviews, fostering a more nuanced comprehension of their experiences and perceptions concerning hospitality services during the Hajj season.

### 3.2 New regularization

Developing a robust machine learning model that can perform well on both training and new testing data poses a significant challenge. Overfitting is a common problem that can impede the efficacy of machine learning techniques. This occurs when the model becomes overly complex due to many training parameters and noise in the input data is used as distinct features to differentiate between different attack classes. To mitigate prediction model error and address this issue, regularization techniques can be employed. However, traditional regularization techniques, such as L1 and L2 regularizers, have limitations that limit their effectiveness. L1 is commonly utilized for feature selection, while L2 assigns less weight to insignificant features. Nevertheless, neither of the methods consider the interdependencies between weight matrix entries. To overcome such limitation, we propose a novel regularizer method that is based on standard deviation and can efficiently manage the dispersion of weight values. This approach prevents the learning model from using a wide range of weight space by multiplying the standard deviation of the weight matrix by its parameter to obtain its regularization term. The standard deviation measures the spread of the weight values within the matrix and is computed by taking the square root of the variance of the weights [[31], [32], [33], [34], [35]].

(1) λσ(w)

The variance of weighted values can be represented by the term σ (standard deviation). This term is added to the loss function of the model to guide the learning process and prevent overfitting. By acting as a penalty, the regularization term encourages the model to use weight values that are more consistent and less scattered across the matrix. This approach aims to create a more stable and robust model that performs well on both training and testing data.

(2) min_(w)={f(x,y:w)+λσ(w)

**Fig. 3:**
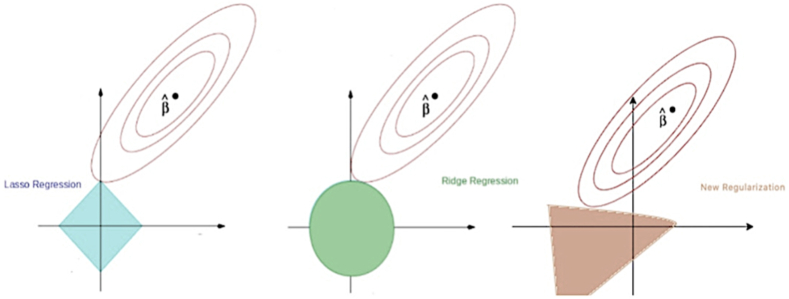
Contours of L1, L2, and new regularizers.

To control the values of the weight matrix, we utilize a parameter λ. We aim to minimize the loss function in regard to (w) by using the standard deviation to adopt values within a specific range. This approach limits the weight space range that the learning model can utilize and considers the standard deviation of the weight matrix, as illustrated in Fig. 3. This can effectively improve the model's performance by handling new and unseen data more efficiently, making it more robust.

### 3.3 Expectation-confirmation theory

The entire mechanism for incorporating Expectation-confirmation theory [36] concepts in this study is to use them as independent variables for the purpose of creating satisfaction, defining expectations (the pre-experience), and measuring satisfaction after receiving the service (the confirmation).

### 3.4 Pre-experience variables

Prior to providing a service, the hospitality industry must assess the expectations of the traveler. This study identifies the hotels that cater to the pilgrim experience in terms of hotel services. There exist a variety of potential concepts regarding pilgrim expectations for hotel services. Various information sources (such as economic factors, national infrastructure, the social environment, the number of reviews, and hotel prices) are a significant factor in influencing pilgrim expectations. Therefore, pilgrims tend to make reservations based on the information provided by the hotel, thereby forming expectations [37].

### 3.5 Post-experience variables

The online review comment is the most important factor and a significant source for identifying different pilgrim perspectives. Common, suggestive, and comparative elements can be identified in the comments. Pilgrim feedback is also comparative in nature because it is frequently compared to the pilgrims' own expectations and experiences to determine the level of satisfaction. In this study, we compare the frequency with which specific terms, such as “expected” or “anticipated,” accompany pilgrim opinions expressed in hospitality industry reviews.

In our analysis, we considered post-experience variables, which included pilgrim comments and subsequent bookings from Bookings.com. To assess overall user satisfaction, we utilized regression analysis. To derive numerical values for analysis, we employed NLP techniques, specifically sentiment analysis and topic modeling. These techniques allowed us to obtain normalized scores ranging from −1 (indicating a negative sentiment) to 1 (indicating a positive sentiment). Subsequently, we transformed these scores into binary labels for classification purposes. Scores equal to or greater than 0.05 were categorized as positive, while scores lower than 0 were categorized as negative. This classification approach provided us with a structured way to gauge user satisfaction based on their comments. In light of this, the study includes pilgrim reviews and online comments containing these terms, which indicate the customer confirmation levels. Furthermore, the emotional components of both positive and negative pilgrim reviews reveal the pilgrim's experience with a specific service [37].

Before pilgrims receive the hospitality service, they form specific expectations (E) regarding the quality and nature of services they anticipate during their pilgrimage. These expectations are influenced by various factors, including economic conditions, national infrastructure, the social environment, reviews, and hotel prices. We mathematically denote pre-experience expectations as the variable E.

Following the completion of their pilgrimage, pilgrims evaluate the perceived service performance (P) based on their actual interactions and experiences with the provided hospitality services. This post-experience evaluation entails a comparison between the services received and the initial expectations. The perceived service performance (P) is a crucial post-experience variable assessed in our research.

To quantify the impact of the service experience, we introduce the concept of confirmation or disconfirmation (C) of pre-experience expectations. This is calculated by taking the difference between the perceived service performance (P) and the initial expectations (E), represented as C=P - E. Positive values of C indicate that the actual service performance surpasses the initial expectations, while negative values of C imply that the performance falls below the expectations.

Pilgrims' overall satisfaction (S) with the hospitality services is directly influenced by the degree of confirmation or disconfirmation (C) experienced. To elucidate this relationship, we derive the mathematical model for pilgrims' satisfaction as follows:

Let us define the function f(C) to represent the impact of confirmation or disconfirmation on pilgrims' satisfaction. Based on the ECT, we assume that the function f(C) is continuous and monotonically increasing. Thus, positive values of C lead to higher satisfaction levels (S) as the perceived service performance exceeds expectations. Conversely, negative values of C result in lower satisfaction levels (S), indicating that the service performance falls short of expectations.

Hence, the mathematical representation of pilgrims' satisfaction (S) can be expressed as: S = f(C).

Through this analytical derivation, we establish the relationship between pilgrims' satisfaction and the confirmation or disconfirmation of their pre-experience expectations. This model enables a comprehensive analysis of the factors influencing pilgrims' satisfaction and offers practical recommendations to enhance the quality of hospitality services and optimize pilgrim experiences.

By incorporating the pre-experience expectations (E), perceived service performance (P), confirmation or disconfirmation (C), and satisfaction (S) within the ECT framework, we establish a robust analytical model for exploring pilgrims' satisfaction with hospitality services. This model allows us to gain valuable insights into how pre-experience expectations influence post-experience evaluations and how meeting or exceeding expectations impacts overall satisfaction during the pilgrimage. The proposed framework enables a comprehensive analysis of the factors influencing pilgrims' satisfaction, offering practical recommendations to enhance the quality of hospitality services and optimize pilgrim experiences.

We assume a hypothetical satisfaction index (S) which determines the overall pilgrims' satisfaction. The extent to meeting or exceeding the pilgrims’ expectations is quantified by an expectation confirmation index (E). The value of E ranges from 0 to 1, where ‘0′ indicates that expectations are completely unmet, whereas we demonstrate that expectations are fully met or even exceeded. Therefore, the relationship between ‘S' and ‘E' can be denoted by the following mathematical linear expression, as an analytical derivation:

S = αE + β, where.

S= Overall satisfaction.

E = Expectation confirmation index

α = Relationship strength between satisfaction and expectation confirmation

β = Intercept term (shows baseline satisfaction where there is zero expectation confirmation).

Analysis of parameters α and β;

α: this parameter denotes change in S for every unit change in E. When α is positive, it means that there is an increase in E, hence a corresponding increase in overall satisfaction (S). On the other hand, a negative α indicates a decrease in E leading to a corresponding decrease in S, which mean that expectations are not met.

β: this denotes the level where expectations of the pilgrims failed to be met.

Linear regression is used to empirically estimate the values of the parameters after collecting data from the pilgrims and measuring their expectation confirmation and overall satisfaction. This can be illustrated using the following pair of data points (see Table 2):

**Table 2:** Data points: pilgrim expectation confirmation and satisfaction.

E	0.2	0.4	0.6	0.8	1.0
S	3.0	3.8	4.0	4.6	5.1

Using Python for linear regression, the following results are obtained:

α = 2.60

β = 2.50.

**Fig. 4:**
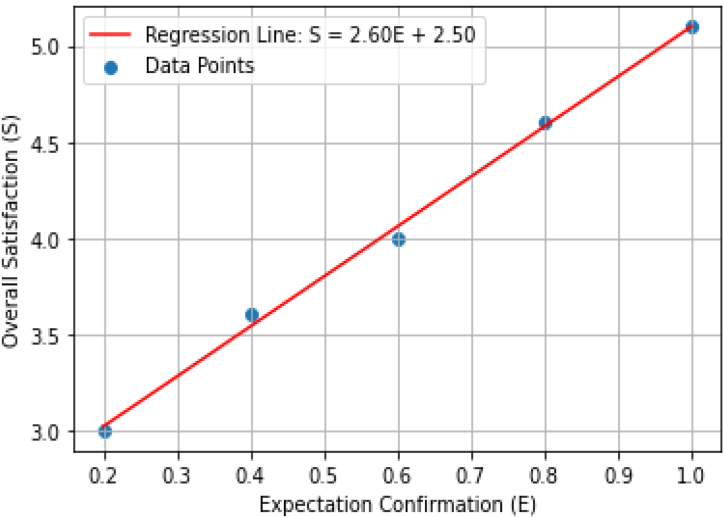
Relationship between expectation confirmation and overall satisfaction.

The graphically representation is as shown in Fig. 4.

**Fig. 5:**
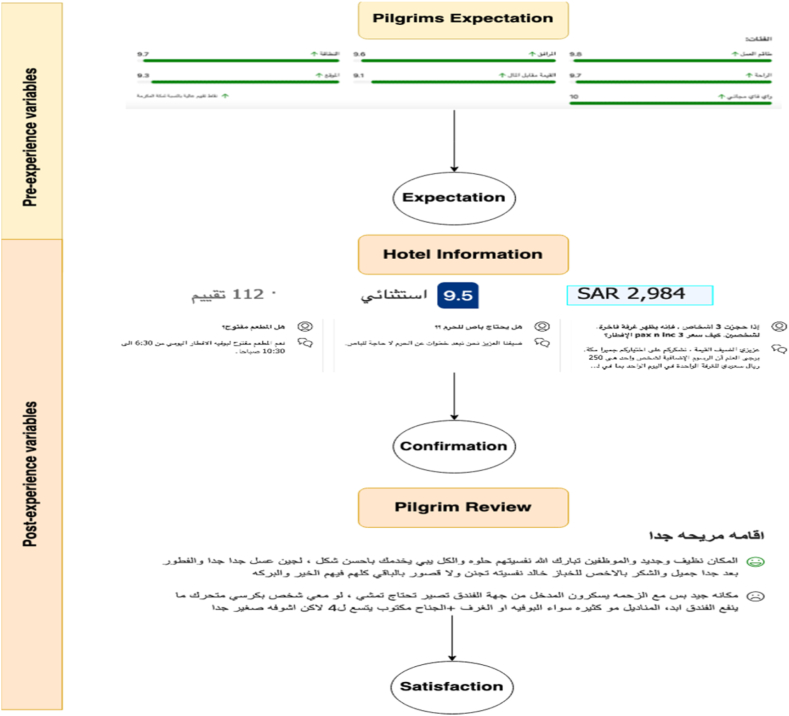
Relationship between expectation confirmation and overall satisfaction.

Through this analytical derivation, we establish the relationship between pilgrims' satisfaction and the confirmation or disconfirmation of their expectations. This model enables a comprehensive analysis of the factors influencing pilgrims' satisfaction and offers practical recommendations to enhance the quality of hospitality services and optimize pilgrim experiences (see Fig. 5).

### 3.6 Proposed model

**Fig. 6:**
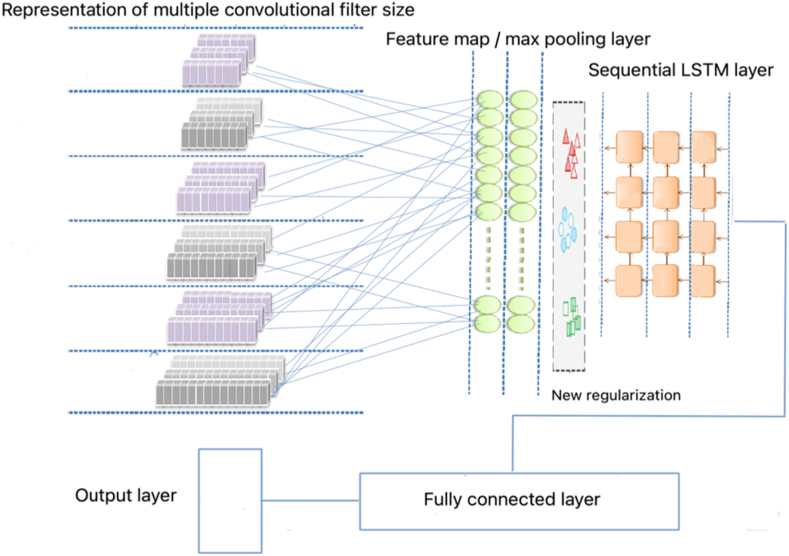
Proposed model architecture.

The prosperity of businesses in the hospitality industry is heavily dependent on the satisfaction of their customers. To measure customer satisfaction, the expectation-confirmation theory is commonly used, which suggests that customers compare their expectations with their actual experiences to determine their satisfaction level with a product or service. In this study, researchers utilized a CNN-LSTM model to investigate the satisfaction levels of pilgrims with hospitality services. To represent words as multidimensional vectors, we used a word embedding model that converts words into sparse vectors, with each word being represented as a unique feature vector in a vector space of a selected dimension. We used AraVec [38] word embedding, which has pre-trained, distributed word representations and encodes semantic and syntactic connections between words. As shown in Fig. 6, the embedding layer was utilized as a pre-processing step to increase the vector representation's density. The CNN model then mapped features by applying a convolutional filter to the input word matrix and using the ReLU activation function to identify aspects related to news. The max-pooling layer sampled the feature maps to make them more resistant to text position changes, while the flatten layer flattened the two-dimensional arrays of the combined feature maps. A fully connected layer changed the output dimension by vector-matrix multiplication, and the output layer utilized the linked layer's output to determine text positivity, using the ADAM optimizer and a sigmoid activation function. By utilizing this model, we were able to investigate pilgrim satisfaction levels accurately and efficiently.

To enhance the accuracy of the CNN-LSTM model, our proposed method integrates the expectation-confirmation theory, which includes pre- and post-experience variables. The input layer is fed with the preprocessed data of pilgrim reviews and ratings, which is then passed to the convolutional layer. Using filters with a 3 × 3 kernel size, the convolutional layer extracts important features from the text data, such as specific words and phrases that reflect pilgrim expectations and satisfaction. The extracted features are summarized and dimensionality-reduced through the pooling layer and then analyzed by the LSTM layer to understand the sequential relationships between the features and their impact on pilgrim satisfaction. The ReLU activation function is utilized to control information flow and ensure the model's predictions are accurate. Furthermore, our model incorporates a new regularization technique to control individual weight values and the relationship between weight matrix entries. This technique multiplies the weight matrix's standard deviation by creating an adaptive weight decay form, thereby guiding the learning process, and preventing the model from taking weight values that are too widely distributed. The regularizer has been extensively tested on various tasks and datasets and proved to be more effective than other regularization methods. Finally, the output layer produces predictions on pilgrim satisfaction based on their expectations and experiences, enabling hospitality businesses to improve their services and enhance customer satisfaction.

### 3.7 Experimental setup

All the experiments in this study were implemented using python programming language. The proposed model was developed using various machine learning and deep learning libraries, such as TensorFlow and Keras. The simulations were conducted on a system with an Intel® CoreTM i3 processor and 20 GB of RAM. Additionally, an NVIDIA GeForce GTX 1080ti graphics processing unit with an 11 GB frame buffer was used to enhance the computational performance of the simulations.

### 3.8 Fine-tuning the proposed model

The Arabic datasets used in this study consist of dialects that vary widely, are not standardized, and are influenced by regional slang. To effectively analyze these dialects, we fine-tuned our proposed model for the downstream task by incorporating annotated data to account for these unique textual features. Firstly, we adjusted our proposed model to generate outputs for classification, then we proceeded to train the model on the annotated dataset until it was sufficiently optimized for sentiment classification. Thus, it allows the model to adapt to the specific characteristics of that dataset and perform better on a given task. In the case of Arabic dialects, fine-tuning a model can help it better understand the nuances of different dialects and improve its performance in sentiment classification. This is because dialects often contain region-specific slang and other language features that can be difficult for a model to understand without prior exposure. By training the model on annotated data, we can help it learn to recognize these dialectal features and improve its performance on the downstream task of sentiment classification.

## 4 Results

This research analyzed the performance of models using the classification report, which contains the main evaluation metrics. The results extracted from the above methods are described through precision, recall, F1-score, and accuracy.

In the results precision (P) represents the rate of predicted positive cases, which are actual positives.

$$P=\frac{\text{True}\;\text{Positive}}{\text{True}\;\text{Positive}+\text{False}\;\text{Positive}}$$

whereas the real positive rate that was also predicted as positive is the recall (R) value.

$$R=\frac{\text{True}\;\text{Positive}}{\text{True}\;\text{Positive}+\text{False}\;\text{Negative}}$$

The F1-score is a biased association of recall and precision, while accuracy is the proportion of correct predictions that has correct positive and negative predicted values.

$$F1=2\;x\frac{P\;x\;R}{P+R}$$

Accuracy is the ratio of the total number of correctly predicted.

$$Accuracy=\frac{\text{True}\;\text{Positive}+\text{True}\;\text{Negative}}{\text{True}\;\text{Positive}+\text{False}\;\text{Positive}+\text{False}\;\text{Negative}+\text{True}\;\text{Negative}}$$

**Fig. 7:**
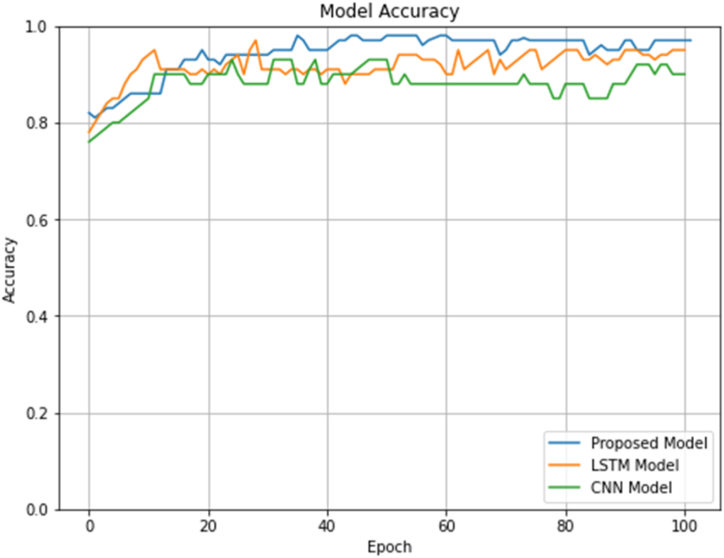
Model accuracy on validation set.

Table 3 provides an overview of the performance of three models, CNN, LSTM, and Proposed, in classifying positive and negative classes based on precision (P), recall (R), F1-score, and accuracy. The results of our study indicate that the proposed model outperformed both the LSTM and CNN models in terms of classification accuracy, precision, recall, and F1-score. Specifically, the proposed model achieved an accuracy of 0.975, which is higher than the accuracy achieved by the LSTM (0.931) and CNN (0.900) models. Moreover, the proposed model achieved a precision score of 0.976 and a recall score of 0.976, both of which are higher than the scores achieved by the other two models. In terms of the F1-score, the proposed model achieved a score of 0.978, while the LSTM and CNN models achieved scores of 0.914 and 0.891, respectively (See Fig. 7). These results indicate that the proposed model is better at classifying the dataset into positive and negative sentiment categories than the LSTM and CNN models. The results show that the proposed model outperformed the other two models in terms of accuracy, precision, recall, and F1-score. This can be attributed to the proposed model's ability to correctly identify positive instances while minimizing the number of false negatives. The high performance of the proposed model can be attributed to its ability to leverage the expectation-confirmation theory using a deep learning model, which allows it to accurately classify Hajj-related posts based on the quality of hospitality services provided.

**Table 3:** Evaluation metrics of the proposed model.

Model	Class	P	R	F1-score	Accuracy
CNN	Positive Negative	0.87527 0.93756	0.92758 0.89161	0.90067 0.91401	0.90
LSTM	Positive Negative	0.90021 0.96927	0.96399 0.91401	0.93101 0.94083	0.95
Proposed model	Positive Negative	0.97613 0.97621	0.97402 0.97815	0.97508 0.97718	0.97

**Fig. 8:**
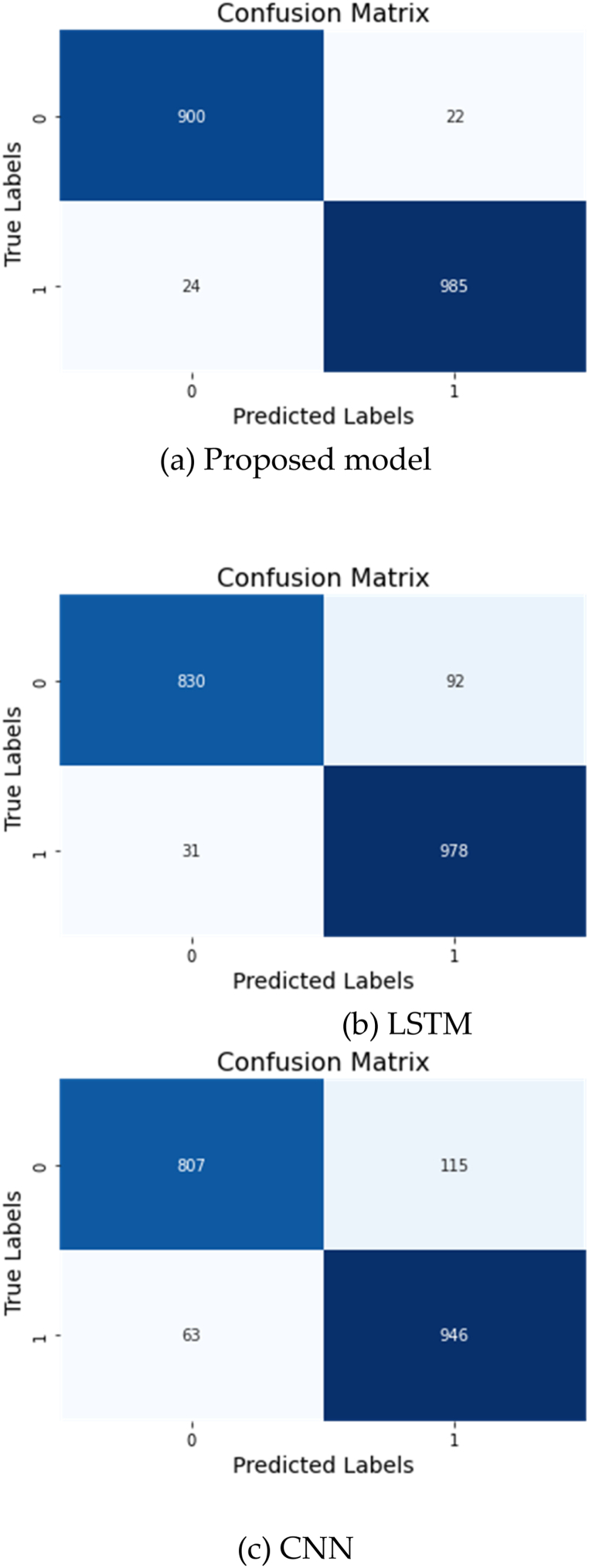
Confusion matrix of all models.

Fig. 8 presented confusion matrix for the proposed model, LSTM model, and CNN model. From the matrix, it can be observed that the proposed model has the highest accuracy score of 0.97, followed by the LSTM model with a score of 0.95 and the CNN model with a score of 0.90. The proposed model was able to correctly classify 985 out of 1009 instances, with only 24 false negative classifications. The LSTM model, on the other hand, correctly classified 978 out of 1009 instances. Additionally, the CNN model classified 946 out of 1009 instances correctly. It is worth noting that the proposed model had a significantly higher precision score of 0.97 compared to 0.96 for the LSTM model and 0.93 for the CNN model. The precision score measures the ability of the model to accurately classify positive instances, and in this case, the proposed model has a higher precision score, indicating that it is better at identifying positive instances with fewer false positives. Furthermore, the F1-score, which is a weighted average of the model's precision and recall, was highest for the proposed model at 0.97, followed by the LSTM model at 0.93 and the CNN model at 0.90. This indicates that the proposed model has a higher balance between precision and recall compared to the other models.

In conclusion, the results of this study demonstrated that the proposed model, which incorporated a novel regularization technique, performed better than the LSTM and CNN models in classifying positive and negative tweets. In particular, the proposed model was better able to deal with overfitting and generalize to unseen data, resulting in a higher F1-score and greater accuracy. These outcomes demonstrate the potential utility of the proposed model for a variety of applications, including brand monitoring, public opinion analysis, and social listening. The incorporation of the new regularization technique enabled the proposed model to more accurately capture the data's underlying features, resulting in enhanced classification performance.

## 5 Discussion

The hospitality industry can benefit from the results of the proposed CNN-LSTM model based on new regularization for predicting customer satisfaction. The model offers several advantages. Firstly, the model can process large amounts of data efficiently, identifying patterns and relationships in a quick and accurate manner. This provides valuable insight into customer expectations and satisfaction, allowing hospitality businesses to improve their services and the overall customer experience. Secondly, the proposed model is flexible, as it can be used to evaluate customer satisfaction in various hospitality settings, such as hotels, restaurants, and resorts. Finally, incorporating pre- and post-experience variables and other factors such as customer demographics and contextual information improves the accuracy of the model and provides a deeper understanding of customer expectations and satisfaction. In conclusion, the use of the CNN-LSTM model with the new regularization technique can aid hospitality businesses in making informed decisions about their services and strategies by gaining a better comprehension of their customers' requirements and preferences. The outcomes of this study have several important implications. Firstly, it demonstrates that the study's design, which utilizes psychological theory and large amounts of data, is more effective than traditional empirical studies. The study also presents a framework for implementing a deep learning model efficiently. The application of experience design and co-creation theory (ECT) in this study allowed for access to sources of information that reflect the customer experience and are closely related to customer behavior. The ECT methodology used in this study highlights the processes that shape and influence the customer experience through the utilization of hotel services. Previous studies in the hotel industry have utilized ECT approaches, but these have been limited by small sample sizes and survey-based research designs [39,40]. In comparison, this study provides a comprehensive understanding of customer expectations, the resulting experiences of hotel service utilization, and the phases of ECT. By employing a CNN-LSTM model with a novel regularization technique, this study contributes both practical and academic research to the understanding of the consumer service experience utilizing multiple sources of information. This study presents a valuable addition to the literature on customer experience in the hotel industry.

## 6 Limitations

The current study gives significant findings; however, several limitations of this study can be attributed to various factors that drive pilgrims' expectations, satisfaction, or confirmation that are not highlighted in this study. For instance, electronic word of mouth was impactful on customer expectations, according to the studies by Yang et al. [41] and Ma et al. [42]. Moreover, socio-demographic details of customers and previous experiences regarding hotel services may influence the level of their satisfaction. On the other hand, rapidly advancing deep learning technologies can develop comprehensive and well-organized prediction models for perceived customer satisfaction from hotel services. The data distribution changes over time, and stakeholders must opt for the distribution of data sets to be trained prior to deploying models. Furthermore, the study analyzes the robustness of the model by integrating a psychological theory into the DL model [43]. Therefore, future research may address the above-mentioned limitations to better understand customer behavior. In addition to the limitations mentioned in the study, there are several other factors that may affect customer expectations, satisfaction, and confirmation. For example, the quality and availability of hotel services, such as food, amenities, and staff responsiveness, may play a role in customer satisfaction. Additionally, the physical environment of the hotel, including its location, cleanliness, and overall appearance, may also impact customer satisfaction. In addition, the availability of alternative accommodation options, such as Airbnb or other short-term rental options, may affect customers' expectations and decision-making process when choosing a hotel. Furthermore, the overall economic climate and travel trends may also play a role in customer behavior. While the study incorporates a psychological theory into the DL model, future research could further explore the integration of psychological theories and models in predicting customer behavior. This could potentially provide a more comprehensive and nuanced understanding of customer satisfaction and expectations. Overall, while this study provides significant findings, there are several limitations that should be considered when interpreting the results and applying them in practice. Future study should address these limitations to better understand customer behavior and satisfaction in the hotel industry.

## 7 Conclusion

In this study, we introduced a deep learning model to delve into the satisfaction levels of pilgrims with hospitality services during Hajj. Our model employed data derived from Hajj-related posts on hotel review websites, incorporating elements of the expectation-confirmation theory. To scale its effectiveness, we conducted a thorough evaluation alongside two other models, CNN and LSTM, with a primary focus on essential metrics such as accuracy, precision, recall, and F1-score. The findings unequivocally demonstrate the superior performance of our proposed model, consistently outperforming the other two models across all evaluation metrics. Notably, the precision for the positive class reached an impressive 0.976, while the recall for the negative class stood at a commendable 0.978. Both classes achieved F1-scores of 0.975 and 0.977, respectively, resulting in an impressive overall accuracy rate of 0.97. The enhanced performance of our proposed model can be attributed to the innovative regularization technique we introduced, which effectively mitigated overfitting concerns and confined the learning model to a more controlled weight space. These compelling results underscore the immense potential of amalgamating deep learning with psychological theory, providing invaluable insights into the intricate factors influencing pilgrims' satisfaction during Hajj.

Moreover, the application of big data and AI technologies demonstrates considerable potential in pinpointing precise areas for improvement within the domain of hospitality services, enabling their alignment with the distinct requirements of pilgrims. As we peer into the horizon of future research initiatives, the integration of advanced Arabic text sentiment analysis tools emerges as a compelling avenue, offering the prospect of further enhancing the comprehensive performance of our model.

## Funding

The authors would like to thank the Deanship of Scientific Research at 10.13039/501100006701Umm Al-Qura University for supporting this work by Grant Code: (22UQU4400257DSR01).

## Data availability statement

Datasets used to support the findings will be made available on reasonable request, subject to approval and adherence to data protection regulations.

## CRediT authorship contribution statement

**Marwan Albahar:** Writing – review & editing, Writing – original draft, Visualization, Investigation, Funding acquisition, Formal analysis, Data curation, Conceptualization. **Foziah Gazzawe:** Writing – review & editing, Writing – original draft, Visualization, Software, Project administration, Methodology, Formal analysis. **Mohammed Thanoon:** Writing – original draft, Software, Resources, Conceptualization. **Abdulaziz Albahr:** Writing – review & editing, Validation, Resources.

## Declaration of competing interest

The authors declare that they have no known competing financial interests or personal relationships that could have appeared to influence the work reported in this paper.
